# Glibenclamide for the Treatment of Acute CNS Injury

**DOI:** 10.3390/ph6101287

**Published:** 2013-10-11

**Authors:** David B. Kurland, Cigdem Tosun, Adam Pampori, Jason K. Karimy, Nicholas M. Caffes, Volodymyr Gerzanich, J. Marc Simard

**Affiliations:** 1 Department of Neurosurgery, University of Maryland School of Medicine, Baltimore, MD 21201, USA; E-Mails: kurland.davidb@gmail.com (D.B.K.); cigdemtosun@gmail.com (C.T.); Adam.Pampori@som.umaryland.edu (A.P.); jkkarimy@gmail.com (J.K.K.); Nicholas.Caffes@som.umaryland.edu (N.M.C.); vgerzanich@smail.umaryland.edu (V.G.); 2 Department of Pathology, University of Maryland School of Medicine, Baltimore, MD 21201, USA; 3 Department of Physiology, University of Maryland School of Medicine, Baltimore, MD 21201, USA

**Keywords:** glibenclamide, Sur1-Trpm4 channel, cerebral ischemia, traumatic brain injury, spinal cord injury, encephalopathy of prematurity, metastatic brain tumor

## Abstract

First introduced into clinical practice in 1969, glibenclamide (US adopted name, glyburide) is known best for its use in the treatment of diabetes mellitus type 2, where it is used to promote the release of insulin by blocking pancreatic K_ATP_ [sulfonylurea receptor 1 (Sur1)-Kir6.2] channels. During the last decade, glibenclamide has received renewed attention due to its pleiotropic protective effects in acute CNS injury. Acting via inhibition of the recently characterized Sur1-Trpm4 channel (formerly, the Sur1-regulated NC_Ca-ATP_ channel) and, in some cases, via brain K_ATP_ channels, glibenclamide has been shown to be beneficial in several clinically relevant rodent models of ischemic and hemorrhagic stroke, traumatic brain injury, spinal cord injury, neonatal encephalopathy of prematurity, and metastatic brain tumor. Glibenclamide acts on microvessels to reduce edema formation and secondary hemorrhage, it inhibits necrotic cell death, it exerts potent anti-inflammatory effects and it promotes neurogenesis—all via inhibition of Sur1. Two clinical trials, one in TBI and one in stroke, currently are underway. These recent findings, which implicate Sur1 in a number of acute pathological conditions involving the CNS, present new opportunities to use glibenclamide, a well-known, safe pharmaceutical agent, for medical conditions that heretofore had few or no treatment options.

## 1. Introduction

Glibenclamide (US adopted name, glyburide) is a member of the sulfonylurea class of drugs and has been in clinical use as an oral hypoglycemic agent since the 1960s [1]. Sulfonylurea drugs all work via a similar mechanism—inhibition of sulfonylurea receptor 1 (Sur1). Patients with diabetes mellitus type II (DM II) benefit from glibenclamide treatment, which inhibits K_ATP_ (Sur1-Kir6.2) channels in pancreatic β islet cells and leads to increased insulin release [2,3,4,5]. Glibenclamide has contributed significantly to the successful treatment of DM II [3], and has provided the foundation upon which newer diabetic mono- and combined therapies have been developed [6].

During the last decade, glibenclamide has received renewed attention due to its pleiotropic protective effects in acute CNS injury. In the CNS, glibenclamide exerts its effects primarily via inhibition of the recently characterized Sur1-Trpm4 channel [7] [(formerly, the Sur1-regulated non-selective cation (NC_Ca-ATP_) channel]. Inhibition of Sur1 with glibenclamide has been found to be an effective treatment in rodent models of various CNS pathologies, including ischemic [8,9,10,11,12,13,14,15] and hemorrhagic stroke [16,17], traumatic brain injury [18,19], spinal cord injury [20,21,22,23], neonatal encephalopathy of prematurity [24,25], and metastatic brain tumor [26] (summarized in Table 1).

In addition to laboratory investigations, retrospective studies of patients with DM II suffering from ischemic stroke suggest that being on a sulfonylurea drug and staying on it during hospitalization for stroke improves outcome at the time of discharge, and reduces the incidence of symptomatic hemorrhagic transformation and mortality [15,27,28,29]. Recently, Phase II clinical trials have begun to evaluate an intravenous formulation of glibenclamide in patients with traumatic brain injury [30] and stroke [31,32,33]. The use of glibenclamide for the treatment of acute CNS injury is highly promising.

Recent publications have reviewed the roles of Sur1 [34], Trpm4 [35] and K_ATP_ [36,37,38] channels in CNS injury. However, our purpose here is to take a different perspective and highlight the potential uses for glibenclamide in treating the injured brain. We begin with an overview of the preclinical studies of glibenclamide therapy for specific CNS insults, and conclude with a discussion of the available data from clinical investigations.

## 2. Review

### 2.1. Focal Cerebral Ischemia

Focal cerebral ischemia is associated with progressive microvascular dysfunction that is manifested initially by the formation of ionic edema, which may be followed by formation of vasogenic edema and, if severe enough, may be followed by the catastrophic structural failure of capillaries—so-called, “hemorrhagic transformation” of an ischemic stroke [39]. Edema in all forms, as well as hemorrhagic transformation, can lead to secondary injury by compressing adjacent tissue. In the most severe cases, malignant cerebral edema and death of the organism may ensue. The molecular events resulting in these various forms of microvascular dysfunction are complex, but they include the upregulation of Sur1 and subsequent Sur1-mediated microvascular dysfunction, as suggested by the salutary effects of inhibiting Sur1 with glibenclamide [34].

**Table 1:** Targets of glibenclamide.

Preclinical Model	Target	citations
**Ischemic stroke**	Edema/Swelling	[8,9,10,11,12]
	Infarct size	
	Neurological function	
	Death	
	Microglial activation	[13,14]
	Neurogenesis/angiogenesis	[14]
**Ischemic stroke with rtPA**	MMP-9 activation	[15]
	Hemorrhagic transformation	
	Neurological function	
	Death	
**Subarachnoid hemorrhage**	BBB permeability	[16]
	Inflammation	
	Caspase-3 cleavage	
	Transynaptic neuronal injury	[17]
	Venous congestion	
	Cognitive function	
**Traumatic brain injury**	Hemorrhagic progression of contusion	[18]
	Capillary fragmentation	
	Neurological function	
	Caspase-3 cleavage	[19]
	Cognitive function	
**Spinal cord injury**	Progressive hemorrhagic necrosis	[20,21,22,23]
	Capillary fragmentation	
	Neurological function	
**Encephalopathy of prematurity**	Venous hemorrhage	[24]
	Brain development	
	Neurological function	
	Hypoxic/ischemic injury	[25]
	Neurological function	
**Metastatic brain tumor**	Edema/Swelling	[26]

The effect of glibenclamide was examined in three different models of non-lethal stroke [9]. With a thromboembolic model and a transient middle cerebral artery occlusion (tMCAo) model, rats that were administered glibenclamide with a delay of up to 6 h exhibited reductions in lesion volumes of 53% and 41%, respectively, at 48 h after the insult. Similarly, in a permanent MCAo (pMCAo) model, a 4-h delay in glibenclamide treatment reduced infarct size by 51%. Sur1 expression following MCAo is greatest after 8 h [8], which may account for the favorable effects observed with delayed administration of glibenclamide.

Independent confirmation that glibenclamide exerts a protective effect in a rat model of stroke was reported by Wali *et al*. [12]. They found that glibenclamide-treated rats showed a significant reduction in infarct volume, lower neurological severity scores, and less hemispheric swelling, compared to vehicle-treated rats. Grip strength was decreased significantly in pMCAo rats compared to shams, and was significantly improved by treatment with glibenclamide.

The effect of glibenclamide also was examined in models of lethal stroke with “malignant” cerebral edema. In one model of malignant infarction (embolization/permanent occlusion), inhibition of Sur1 with glibenclamide reduced mortality by 50% [8]. In another model of malignant infarction (6-h occlusion/reperfusion), the efficacy of glibenclamide was compared to decompressive craniectomy (DC) [10]. DC often is performed on patients with severe stroke complicated by “malignant edema” [40]. Glibenclamide significantly reduced the mortality rate to 5%, whereas the vehicle-treated group had 67% mortality at 24 h. Compared to the DC cohort, glibenclamide-treated rats exhibited less brain swelling at 24 h and improved neurological outcome that persisted for the 2 weeks of observation. Both DC and glibenclamide eliminated mortality, but neurological function during the next 2 weeks was significantly better with glibenclamide compared with DC. Deep white matter, as well as watershed cortex between the anterior and middle cerebral artery territories, were significantly better preserved with glibenclamide compared to DC. Overall, the authors concluded that glibenclamide treatment in rats was superior to DC after malignant infarction in rats.

Currently, the only Food and Drug Administration (FDA)-approved drug for the treatment for stroke is recombinant tissue plasminogen activator (rtPA), but its use is associated with an increase in risk for hemorrhagic conversion, due in part to activation of matrix metalloproteinase (MMP) 9 [41,42]. Recent work indicates that glibenclamide inhibits ischemia-induced activation of MMP-9, reduces edema and symptomatic hemorrhagic transformation, and improves preclinical outcomes in MCAo models of stroke with co-administration of rt-PA [11,15].

In acute ischemic injury to the CNS, the principal target of glibenclamide is the Sur1-Trpm4 channel [8], which recently was characterized molecularly [7]. However, Ortega and colleagues [13] proposed that glibenclamide is neuroprotective after cerebral ischemia due to modulation of microglial K_ATP_ (Sur1-Kir6.2) channels. In culture, a proinflammatory stimulus of lipopolysaccharide (LPS) was sufficient for BV2 cells (transformed microglial cell line) to upregulate the K_ATP_ channel subunits, Sur1 and Kir6.2. Adding glibenclamide to the medium augmented the reactive morphology, the phagocytic capacity and the release of cytokines (TNFα). Rats that were treated with glibenclamide following MCAo exhibited reduced subcortical (ventral pallidum) necrosis and neuronal loss. The authors noted that activated microglia in the lesion core specifically expressed Sur1 and Kir6.2, suggesting that neuroprotection mediated by glibenclamide was due, in part, to increased microglial phagocytic capacity after K_ATP_ channel inhibition.

In a second study by this group, Ortega *et al*. [14] analyzed long-term outcomes of glibenclamide treatment administered after focal ischemia in rats. They reported marked improvements in sensorimotor and cognitive functions up to 1 month. Blockade of Sur1 by glibenclamide was accompanied by an increase in BrdU and NeuN labeling in and around the infarct, suggesting an enhancement in neurogenesis in the cortex. These newly dividing neurons have the potential to migrate to the ischemic lesion and modulate repair mechanisms [43,44,45]. In addition, glibenclamide treatment enhanced angiogenesis in the cortex and hippocampus, which is a well characterized hallmark of recovery following ischemic stroke.

As the foregoing illustrates, a considerable number of preclinical studies utilizing a variety of different models and originating from three independent laboratories suggest that glibenclamide has an important role in reducing adverse secondary manifestations and increasing favorable outcomes in rat models of focal cerebral ischemia. The preclinical data support further study of glibenclamide for the treatment of ischemic stroke in humans. Indeed, a clinical trial is underway to investigate whether glibenclamide improves outcomes after large ischemic stroke (see below).

### 2.2. Subarachnoid Hemorrhage

Subarachnoid hemorrhage (SAH) accounts for 10% of the overall stroke burden in society and has a 30-day mortality rate approaching 50% [46]. Those who survive experience secondary injury from numerous injury cascades including oxidative stress, neuroinflammation, and vasospasm [47,48,49]. Therapy aimed at reducing any of these events would bestow a significant benefit on patients suffering from SAH.

The effect of glibenclamide was examined in a model of moderate SAH induced by unilateral filament puncture of the internal carotid artery [16]. Twenty-four hours after injury, SAH caused a large increase in blood-brain-barrier (BBB) permeability [measured as extravasation of immunoglobulin (Ig) G] and disrupted the normal junctional localization of the tight junction protein, zona occludens (ZO) 1. Glibenclamide significantly reduced ZO-1 abnormalities, resulting in less edema formation. In addition, SAH led to large increases in several markers of inflammation, including tumor necrosis factor (TNF) α and nuclear factor (NF) κB, and markers of cell injury or cell death, including IgG endocytosis and cleavage of caspase-3. Glibenclamide significantly reduced these effects as well.

Recently, experiments were conducted to study the effect of glibenclamide following unilateral and bilateral stereotactic injections of autologous blood into the subarachnoid space of the entorhinal cortex. This novel model of SAH was intended to reproduce the effects of high grade SAH involving “eloquent” cortex that often leaves patients with persistent cognitive impairments. Glibenclamide significantly reduced transynaptic apoptosis of hippocampal neurons, reduced venous congestion and, most importantly, significantly ameliorated impairments in spatial learning [17].

### 2.3. Traumatic Brain Injury

In the United States, 1.4 million people are affected by traumatic brain injury (TBI) annually, and there are more than five million Americans who live with physical, cognitive, behavioral, and emotional disabilities resulting from TBI [50]. Cognitive deficits that result from TBI are often persistent, lasting years after the trauma [51].

One of the least studied, yet most sinister aspects of TBI is the spatiotemporal progression (“blossoming”) of a hemorrhagic contusion [52,53,54,55], which has been referred to as “hemorrhagic progression of a contusion” (HPC) [56]. HPC incites further injury to the surrounding tissues due to mass effect and biochemical toxicity induced by various cytokine, chemokine and other pathways. Trauma-related coagulopathy may contribute to HPC, but treatments aimed at clinical or sub-clinical bleeding disorders show only modest treatment effects [57], suggesting that other mechanisms underly HPC.

Utilizing a rat model of focal cortical contusion, it was found that Sur1 was prominently upregulated in penumbral cells and microvessels within hours of injury, and remained elevated up to 24 h after injury [18]. Sur1 expression mirrored the spatial and temporal extent of the hemorrhagic contusion, suggesting that Sur1 upregulation may be associated with HPC.

Glibenclamide treatment administered shortly after injury was highly effective in reducing the pathological sequelae of TBI in these animals [18]. HPC was consistently observed in vehicle-treated controls, but was largely absent in glibenclamide-treated animals, even 24 h after the trauma. Immunolabeling with vimentin showed that glibenclamide treatment prevented capillary fragmentation following contusion. Importantly, glibenclamide significantly reduced lesion size and improved rearing behavior 1 week after the trauma. The observation that glibenclamide reduces brain edema and contusion volume after experimental TBI recently was verified by an independent laboratory [58].

The effect of glibenclamide was examined on performance in the Morris water maze in a cortical impact model of TBI calibrated to avoid hemorrhagic contusion in the hippocampus [19]. Both vehicle-treated and glibenclamide-treated rats were spared gross deficits in spatial memory in this model. However, in a task more sensitive to mild injury, rapid spatial learning was significantly impaired only in vehicle-treated animals. Thus, glibenclamide is neuroprotective even after a modest injury that is specifically designed to avoid hemorrhagic progression of a contusion.

### 2.4. Spinal Cord Injury

As in TBI, trauma-induced spinal cord lesions expand and evolve over time [59,60,61,62], resulting in a phenomenon termed “progressive hemorrhagic necrosis” (PHN) [63,64,65,66,67]. PHN was first recognized over three decades ago, and had long eluded understanding and treatment. PHN is characterized by petechial hemorrhages occurring in tissues adjacent to the primary lesion over 3–24 h following trauma, and eventually coalescing into a characteristic lesion of hemorrhagic necrosis [68,69]. Although the underlying mechanism was unknown, investigators speculated that damage to the endothelium of spinal cord capillaries and postcapillary venules was a major factor in the pathogenesis of PHN [65,70,71].

In a unilateral cervical spinal cord injury (SCI) model, PHN is associated with Sur1 upregulation in capillaries and post-capillary venules in a time-dependent fashion [20]. Immediately after injury, Sur1 immunolabeling is absent, but becomes apparent at 6 h surrounding the necrotic lesion. By 24 h, the necrotic lesion has enlarged and Sur1 has expanded from the rim of the necrotic lesion into tissues distal to the impact site, where it is predominantly associated with capillaries. Sur1 is also present in the core of the hemorrhagic lesion, where it is associated with various cells and structures, including neuron-like and capillary-like structures. Blockade of Sur1 via glibenclamide reduces PHN and improves neurobehavioral assessments, compared to vehicle-treated controls. Glibenclamide eliminates capillary fragmentation and spares contralateral and ipsilateral white matter tracts. Strikingly, at 1–6 weeks, lesion volumes are reduced 2–3-fold compared to controls. The observation that glibenclamide attenuates PHN and improves functional outcomes after experimental SCI has been independently verified by Popovich *et al*. [72].

The Sur1-Trpm4 channel can be targeted not only by glibenclamide but by riluzole as well, since riluzole blocks Trpm4, the pore-forming subunit of the channel [23]. Riluzole currently is in clinical trials for the treatment of acute SCI [73,74]. The effects of glibenclamide *vs*. riluzole were compared after cervical spinal cord injury in rats, with both drugs being administered at the clinically relevant time of 3 h after trauma [23]. Both riluzole and glibenclamide were associated with reduced capillary fragmentation, reduced PHN, reduced neuronal death, and significant recovery of neurological function, compared to controls. However, in tests involving complex neuromotor function, glibenclamide-treated rats performed better than riluzole-treated rats. Glibenclamide also was superior to riluzole in terms of tissue sparing at 6 weeks.

As might be expected, the magnitude of the benefit observed with glibenclamide in SCI depends on the magnitude of the primary injury [22]. However, all studies to date examining functional outcome and lesion size at 6 weeks have demonstrated a significant beneficial treatment effect, regardless of the initial severity.

### 2.5. Encephalopathy of Prematurity

Brain injury due to perinatal brain ischemia and hemorrhage is a major cause of mortality and acute neurological injury in neonates. The constellation of syndromes and symptoms collectively is referred to as “encephalopathy of prematurity” (EP). In infants, injury due to hemorrhagic events (hemorrhagic EP) is a well-known risk factor for cerebral palsy and is often associated with subsequent life-long neurodevelopmental disabilities, including motor, behavioral and cognitive disorders [75,76,77,78]. The specific cellular and molecular mechanisms of this multifaceted injury are poorly understood, which has made it difficult to identify effective therapeutic targets.

In the CNS, ischemia/hypoxia upregulate Sur1-Trpm4 channels in microvascular endothelium, with channel activation being responsible for progressive secondary hemorrhage. In a study of post-mortem tissues from premature infants, Sur1 and its transcriptional antecedent, hypoxia inducible factor (HIF) 1, were found to be prominently upregulated in periventricular tissue and veins in infants who sustained periventricular hemorrhage, but not in controls [79]. These observations make an intriguing case for the potential involvement of Sur1-regulated channels in the pathology of hemorrhagic encephalopathy of prematurity.

Commonly, perinatal hypoxic/ischemic injury is modeled by the Rice-Vanucci method, or modifications thereof [80]. The general procedure to mimic the human condition in rat pups involves postnatal carotid ligation plus exposure for a period of time to an hypoxic environment. Zhou *et al*. [25] employed this model and showed that glibenclamide, administered to pups shortly after the insult and again 24 h later, was neuroprotective after moderate hypoxic/ischemic injury. They observed a decrease in infarct volume, which did not reach statistical significance, but that was reflected in significantly less severe motor impairment in glibenclamide treated animals.

The Rice-Vanucci model does not lead to the hemorrhagic forms of EP that are so common in extremely premature infants. Koltz and colleagues [81] developed a novel rat model of hemorrhagic EP in which in utero ischemia (IUI) is combined with an episode of postnatal raised intrathoracic pressure (RIP), to mimic the effect of mechanical ventilation on central venous pressure. These tandem insults were shown to cause hemorrhages in the periventricular tissues and surrounding white matter tracts that recapitulate the human condition with great fidelity. The pathology observed in this model also is accompanied by developmental delays and long-term vestibulomotor and cognitive impairments.

Recently, Tosun *et al*. [24] showed that in this model of hemorrhagic EP, glibenclamide delivered to the mother at the end of the pregnancy was significantly neuroprotective by several measures. The mortality of pups born from glibenclamide-treated mothers was reduced by over 30%. Additionally, glibenclamide treatment dramatically reduced the incidence and regional extent of postnatal brain hemorrhages—any evidence of bleeding was limited to rare petechial hemorrhages, compared to the substantial venous hemorrhages detected in almost all periventricular areas examined in vehicle treated animals. Glibenclamide treatment of the mother also reduced the number of periventricular ED-1 positive microglia/macrophages. After 7 weeks, motor and cognitive functions were evaluated in a subset of the pups subjected to IUI/RIP. Animals exposed to glibenclamide while in utero performed similar to naïve rats on a narrow beam walk test, whereas those exposed to vehicle while in utero were significantly impaired. Similarly, in a challenging accelerating RotaRod task, the vehicle-exposed group had impairments that were significantly attenuated in the glibenclamide-exposed cohort. In a task of rapid spatial learning in the Morris water maze, glibenclamide-exposed rats exhibited a correct preference for the new quadrant, performing similar to the naïve group, whereas rats from the vehicle group showed no such preference. Strikingly, at the time of euthanasia (P49), total body and brain weights were significantly reduced in pups from vehicle-treated mothers, whereas pups from glibenclamide-treated mothers exhibited significant preservation of body and brain mass, *i.e.*, significantly less developmental delay.

### 2.6. Metastatic Tumor

As reviewed above, glibenclamide is highly effective in reducing edema in the context of cerebral ischemia and subarachnoid hemorrhage. Cerebral edema also is a clinically important problem in the context of brain tumors, including the most common one, metastatic brain tumor. Recently, Thompson *et al*. [26] intracerebrally implanted nude rats with small cell lung carcinoma or melanoma cells, and compared the effects of glibenclamide and dexamethasone on brain tumor barrier (BTB) permeability. Both glibenclamide and dexamethasone decreased BTB permeability in the two metastatic tumor models. Moreover, the effect of both drugs was attributed to a decrease in ZO-1 gap formation, similar to observations reported in SAH [16]. Notably, glibenclamide was at least as effective as dexamethasone, which currently is the standard of care, but can be associated with adverse side effects, especially during prolonged use.

### 2.7. Retrospective Clinical Studies in Stroke

The effect of sulfonylureas on stroke in humans has been examined retrospectively in diabetic patients presenting acutely with ischemic stroke. In these studies, control patients (patients whose diabetes was managed without sulfonylurea drugs) were compared with patients whose diabetes was managed with sulfonylurea drugs and who were maintained on sulfonylurea drugs after hospitalization for stroke.

Kunte and colleagues [27] analyzed the medical records of diabetics who were admitted within 24 h of onset of acute ischemic stroke to the Neurology Clinic, Charité Hospital, Berlin, Germany, from 1994 to 2000. After exclusions, the cohort consisted of 28 control patients not on a sulfonylurea and 33 patients in the treatment group, who were treated with a sulfonylurea (glibenclamide, glimepiride or glibonuride) at admission through discharge. Other than stroke subtype, baseline variables were similar between control and treatment groups. The primary outcome was a decrease in National Institutes of Health Stroke Scale (NIHSS) score of 4 points or more from admission to discharge, or a discharge NIHSS score = 0, which represent a “major neurological improvement”. The secondary outcome, which signifies functional independence, was a discharge modified Rankin Scale (mRS) score of 2 or less. There were striking differences between the control and treatment groups, which were independent of gender, previous transient ischemic attack and blood glucose levels. The primary outcome was reached by 36% of patients in the treatment group and 7% in the control group [odds ratio (OR), 7.4; 95% confidence interval (CI), 1.5–37; *p* = 0.007]. The secondary outcome was reached by 82% *versus* 57% (OR, 3.4; CI, 1.1–11; *p* = 0.035). Among patients with nonlacunar stroke, 42% of patients on sulfonylureas achieved the primary outcome *versus* 0% of controls.

In another retrospective study by Kunte *et al*. [29], a review of medical records was performed on DM II patients admitted to Charité Hospital with an acute ischemic stroke from January 2005 to December 2006. After exclusions, the cohort consisted of 177 control patients not on a sulfonylurea and 43 patients in the treatment group, who were treated with a sulfonylurea (glibenclamide, glimepiride or gliquidone) at admission through discharge. In this study, the primary outcome measure was evidence of symptomatic hemorrhagic transformation prior to discharge or within 21 days of the ischemic stroke, and the secondary outcome measures were any hemorrhagic transformation (symptomatic or asymptomatic), death in hospital, mRS score of 2 or less, and a decrease in NIHSS score of 4 points or more from admission to discharge, or a discharge NIHSS score = 0. Patients treated with a sulfonylurea drug were significantly more likely to survive hospitalization [no patient in the sulfonylurea group died, compared to 18 (10%) in the control group (*p* = 0.027)] and significantly less likely to experience hemorrhagic transformation [20 control patients (11%) experienced symptomatic hemorrhagic transformation, whereas no patient in the sulfonylurea group experienced symptomatic hemorrhagic transformation (*p* = 0.016)]. In agreement with their prior observations [27], patients treated with a sulfonylurea were significantly more likely to have better neurological outcomes. Even after matching for imbalances in baseline variables and excluding outliers, the influence of sulfonylurea treatment remained significant.

The results of another retrospective study were reported at an International Stroke Conference, the data coming from the Registry of the Canadian Stroke Network, and concerning patients presenting to 11 stroke centers in Ontario between 1 July 2003 and 31 March 2008 [28]. Of the 2,448 DM II patients included in the study, 1,469 were in the control group and 729 were in the sulfonylurea group. The likelihood of in-hospital mortality was less for patients on sulfonylurea drugs compared to control patients (OR, 0.51; CI, 0.37–0.71; *p* < 0.05). Sulfonylurea-treated patients also had a lower likelihood of neurological worsening (OR, 0.70; CI, 0.55–0.89; *p* < 0.05). A subgroup of patients was analyzed who were treated with rtPA. For patients who had been on and continued on a sulfonylurea drug, compared with controls not on sulfonylureas, the likelihood of in-hospital mortality was less (OR, 0.30; CI, 0.12–0.73; *p* < 0.05), and the likelihood of neurologic worsening was less (OR, 0.52; CI, 0.28–0.98; *p* < 0.05). The potential for Sur1 inhibition with sulfonylurea drugs to compliment the use of rtPA in cerebral ischemia has been reviewed [15].

In summary, these retrospective studies of diabetic patients presenting with stroke suggest that, if a patient is on a sulfonylurea drug at presentation, this drug should be continued, not stopped, unless a contraindication is identified.

### 2.8. Prospective Clinical Trials

The neuroprotective properties of glibenclamide observed in laboratory experiments and in retrospective human studies have led to the initiation of prospective clinical trials. These prospective trials are evaluating an injectable formulation of glibenclamide (RP-1127; Remedy Pharmaceuticals, Inc., New York, NY, USA) that is being administered IV.

#### 2.8.1. Glyburide Advantage in Malignant Edema and Stroke (GAMES)

Recently, a 2-center, prospective, open label, Phase IIa “pilot” study of RP-1127 was completed (ClinicalTrials.gov Identifier: NCT01268683) [33]. This clinical trial tested the effect of RP-1127 in 10 patients with a severe anterior circulation ischemic stroke (baseline infarct volume ≥ 82 mL; mean, 102 ± 23 mL; baseline NIHSS score, 19 ± 8) at high risk for malignant cerebral edema. Of the 10 patients, one died, even after decompressive craniectomy. The incidence of malignant edema was 20%, compared with 88% in a prospective observational study of patients with infarct volumes ≥82 mL [82]. Moreover, 8/10 patients did not require osmotherapy, intubation, or decompressive craniectomy, and there were no clinically significant parenchymal hematomas (“PH1/PH2”), in contrast to parenchymal hematoma rates of ~30% in the DEFUSE and EPITHET trials in patients with a malignant profile [83]. The proportion of patients with 30-day modified Rankin Scale (mRS) scores ≤4 was 90%, compared with 24% (at 12 months) in control patients from a pooled analysis of decompressive craniectomy trials [84], or 29% (at 90 days) for patients with infarct volumes >70 mL [85].

A larger clinical trial studying the effect of RP-1127 in patients with large ischemic strokes currently is underway and actively recruiting patients (ClinicalTrials.gov Identifier: NCT01794182) [32]. The primary objective is to demonstrate the safety and efficacy of RP-1127 compared to placebo in subjects with a severe anterior circulation ischemic stroke who are likely to develop malignant edema. The primary efficacy outcome is the proportion of patients with a mRS at Day 90 ≤4 without the need for decompressive craniectomy (DC). Secondary efficacy outcomes are the proportion of patients who develop or undergo the following: malignant edema within 7 days, DC within 90 days, early neurological deterioration within 72 h, parenchymal hematomas within 7 days, ipsilateral hemispheric swelling measured by MRI within 96 h, 90-day mRS 0–3 and 0–4, Barthel Index measurements of Activities of Daily Living at 90 days, and all-cause mortality at 90 days.

#### 2.8.2. Glyburide for TBI

A prospective, multicenter, placebo-controlled, double-blind, Phase IIa trial of RP-1127 is underway to test the effect of RP-1127 in patients with moderate-to-severe TBI (ClinicalTrials.gov Identifier: NCT01454154) [30]. The primary outcome measures are change in edema from baseline within 72 h (apparent diffusion coefficient, volume, and free water), change in hemorrhage from baseline within 72 h (hemorrhagic burden index, number of hemorrhagic lesions, size of hemorrhagic lesions) and safety through 180 days.

### 2.9. CNS Targeting of Glibenclamide

Normally, glibenclamide and other sulfonylureas do not accumulate in the brain in physiologically meaningful concentrations [86], accounting for the observation that, despite decades of clinical use in diabetic patients, there are no reports of side effects involving the CNS that are attributable directly to sulfonylureas (as distinct from CNS effects of hypoglycemia), even though the brain contains many regions with neurons that express K_ATP_ channels [34,87]. However, penetration of glibenclamide into the CNS is enhanced after injury, either ischemic or traumatic. The combination of blood-brain barrier (BBB) breakdown and the relatively low pH environment that occurs after an insult to the brain has the effect that a weak acid such as glibenclamide is “selectively” taken up by injured CNS tissues while being excluded from normal CNS tissues. Studies using the fluorescent analog BODIPY-glibenclamide, as well as [3H] glibenclamide, have shown that glibenclamide is indeed transported preferentially into the ischemic brain [8,13]. As a result, relatively low doses of drug can be used to obtain a favorable therapeutic effect, while minimally affecting insulin secretion in the pancreas [34] and minimizing potential side effects.

## 3. Conclusions

The pleiotropic neuroprotective effects of glibenclamide have been well-substantiated over the past decade in clinically relevant rodent models of human disease. Acting via inhibition of the Sur1-Trpm4 and K_ATP_ channels (and perhaps other mechanisms), glibenclamide protects microvascular endothelium to reduce the formation of edema and secondary hemorrhage, it inhibits neuronal cell death, it exerts potent anti-inflammatory effects and it promotes neurogenesis. Retrospective studies of diabetics, as well as a recent Phase IIa pilot study in nondiabetic patients, suggest a highly promising translational potential for therapeutic intervention with glibenclamide in ischemic stroke. Studies focusing on ischemic stroke as well as the ongoing clinical trial in TBI underscore the impact that glibenclamide may have in the treatment of ischemic, traumatic, and inflammatory injury to the CNS.

Glibenclamide was developed a half-century ago, and significantly impacted the health of countless diabetics before newer agents largely displaced its use. As we suggest here, the use of glibenclamide may be on the brink of a resurgence for the treatment of acute CNS injury. If the preclinical and early phase clinical work continues to show promising results, it is likely that glibenclamide will become a safe and viable therapeutic option for patients suffering from a host of devastating CNS injuries. The scope of potential impact is difficult to overstate.
